# Optimizing 1D ^1^H-NMR profiling of plant samples for high throughput analysis: extract preparation, standardization, automation and spectra processing

**DOI:** 10.1007/s11306-019-1488-3

**Published:** 2019-02-26

**Authors:** Catherine Deborde, Jean-Xavier Fontaine, Daniel Jacob, Adolfo Botana, Valérie Nicaise, Florence Richard-Forget, Sylvain Lecomte, Cédric Decourtil, Kamar Hamade, François Mesnard, Annick Moing, Roland Molinié

**Affiliations:** 1 UMR1332 Biologie du Fruit et Pathologie, Centre INRA de Nouvelle Aquitaine Bordeaux, INRA, Univ. Bordeaux, av Edouard Bourlaux, 33140 Villenave d’Ornon, France; 2 Plateforme Métabolome du Centre de Génomique Fonctionnelle Bordeaux, MetaboHUB, IBVM, Centre INRA de Nouvelle Aquitaine Bordeaux, av Edouard Bourlaux, 33140 Villenave d’Ornon, France; 3 BIOPI - EA 3900, Univ. Picardie Jules Verne, 1, rue des Louvels, 80037 Amiens Cedex, France; 4 JEOL UK, Silver Court, Watchmead Road, Welwyn Garden City, AL7 1LT UK; 5 UR1264 MycSA, INRA, Centre INRA de Nouvelle Aquitaine Bordeaux, av Edouard Bourlaux, 33140 Villenave d’Ornon, France

**Keywords:** Metabolomics, NMR acquisition, NMR spectra processing, Sample preparation

## Abstract

**Introduction:**

Proton nuclear magnetic resonance spectroscopy (^1^H-NMR)-based metabolomic profiling has a range of applications in plant sciences.

**Objectives:**

The aim of the present work is to provide advice for minimizing uncontrolled variability in plant sample preparation before and during NMR metabolomic profiling, taking into account sample composition, including its specificity in terms of pH and paramagnetic ion concentrations, and NMR spectrometer performances.

**Methods:**

An automation of spectrometer preparation routine standardization before NMR acquisition campaign was implemented and tested on three plant sample sets (extracts of durum wheat spikelet, *Arabidopsis* leaf and root, and flax leaf, root and stem). We performed ^1^H-NMR spectroscopy in three different sites on the wheat sample set utilizing instruments from two manufacturers with different probes and magnetic field strengths. The three collections of spectra were processed separately with the NMRProcFlow web tool using intelligent bucketing, and the resulting buckets were subjected to multivariate analysis.

**Results:**

Comparability of large- (*Arabidopsis*) and medium-size (flax) datasets measured at 600 MHz and from the wheat sample set recorded at the three sites (400, 500 and 600 MHz) was exceptionally good in terms of spectral quality. The coefficient of variation of the full width at half maximum (FWHM) and the signal-to-noise ratio (S/N) of two selected peaks was comprised between 5 and 10% depending on the size of sample set and the spectrometer field. EDTA addition improved citrate and malate resonance patterns for wheat sample sets. A collection of 22 samples of wheat spikelet extracts was used as a proof of concept and showed that the data collected at the three sites on instruments of different field strengths and manufacturers yielded the same discrimination pattern of the biological groups.

**Conclusion:**

Standardization or automation of several steps from extract preparation to data reduction improves data quality for small to large collections of plant samples of different origins.

**Electronic supplementary material:**

The online version of this article (10.1007/s11306-019-1488-3) contains supplementary material, which is available to authorized users.

## Introduction

NMR metabolomic profiling has been widely used in the plant sciences (Deborde et al. 2017; Kim et al. 2010; Le Gall et al. 2017; Moing et al. 2004; Rivas-Ubach et al. 2013; Ward et al. 2007). Its advantages are the universal detection of organic compounds with a high dynamic range, a good reproducibility, a relatively simple implementation for the screening and quantification of a range of major metabolites, and a provision of structural information for compound identification. However, a major drawback is its low sensitivity. The scientific domains for which plant NMR metabolomic profiling has been used include pharmacology, plant food or feed characterization, the study of abiotic or biotic stresses including plant-pathogen or plant-pest relationships, plant functional genomics and green biotechnology. Although liquid or high resolution magic angle spinning (HR-MAS) NMR can be used for the metabolomic profiling of plant samples (e.g. Corsaro et al. 2015; Flores et al. 2019), we focus here on liquid NMR of plant extracts. Liquid ^1^H-NMR can be used for untargeted metabolomic profiling or for semi-targeted analyses with relative quantification of spectral regions or absolute quantification of selected compounds (Allwood et al. 2011).

**Fig. 1:**
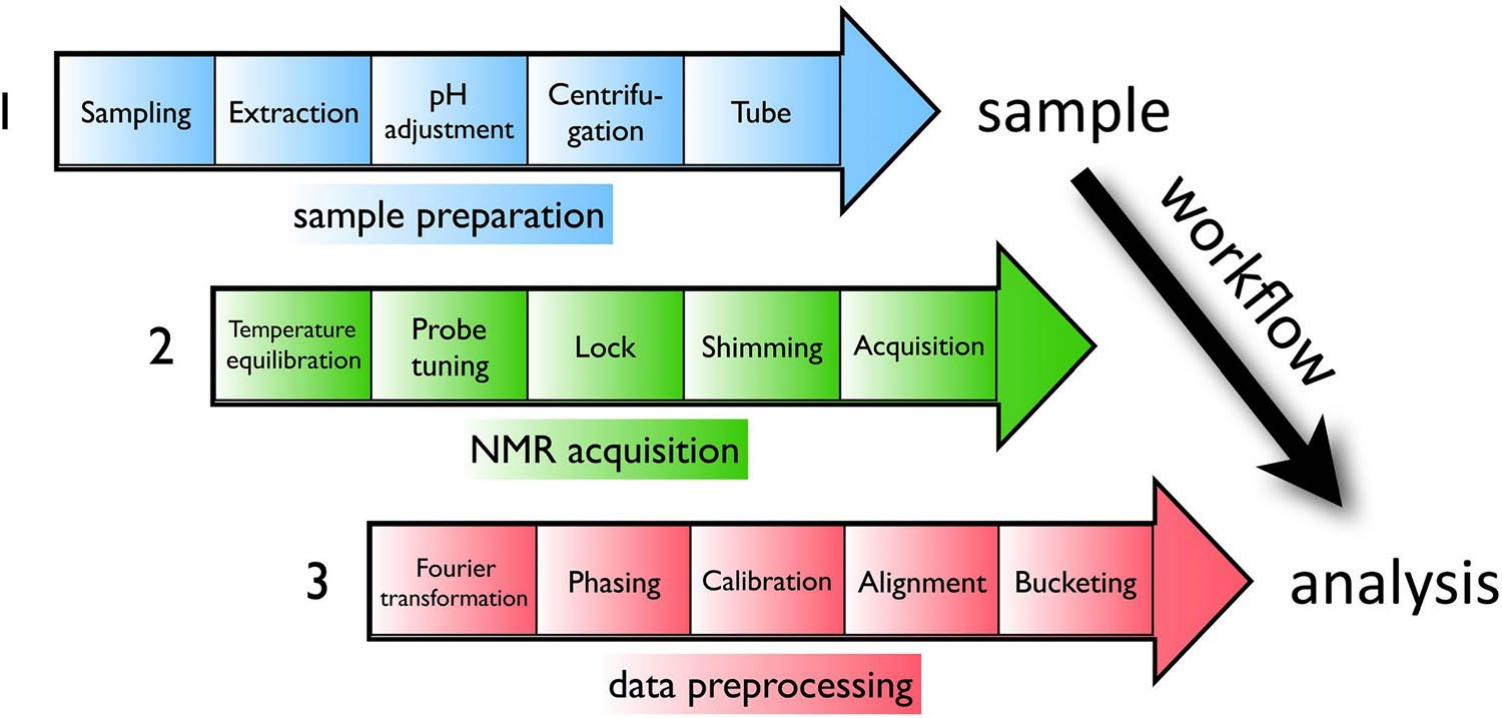
^1^H-NMR metabolomic workflow of plant semi-polar extracts. 1, sample preparation; 2, NMR acquisition; 3, data pre-processing

An ^1^H-NMR metabolomic workflow begins after the harvest of plant samples and consists in four steps: sample and extract preparation, spectra acquisition, spectra and data processing and metabolite identification (Fig. 1). The steps covered in this tutorial range from extract preparation to data processing. The samples are usually frozen in liquid nitrogen and freeze-dried to stop metabolism. Samples should preferentially be stored at − 80 °C, ideally for less than 6 months. Their stability during storage needs to be assessed. According to the number of samples, three categories of sample series can be defined: small—(fewer than 50 samples), average—(50 to 200 samples) and large-size (over 200 samples). Ideally, step 1 (sample preparation) and step 2 (data acquisition) should be done over a short period of time (1 to 2 weeks), and preferably continuously (less than 1-month gap). The samples must be analyzed randomly (Defernez and Colquhoun 2003). Concerning extraction, polar and especially semi-polar extracts are widely used in order to obtain compositional information about major compounds such as organic acids, carbohydrates, amino acids, quaternary ammonium compounds, biogenic amines and hydroxycinnamic acids or esters (Allwood et al. 2011; Baker et al. 2006). Apolar extracts may also be of interest for plant biochemical phenotyping using ^13^C or ^1^H-NMR (Maulidiani et al. 2018; Palomino-Schätzlein et al. 2011) but are not considered in the present study. Semi-polar methanolic extracts are a good compromise for accessing both major semi-polar primary and specialized metabolites (Kim et al. 2010), but specific adaptations to plant samples are often required to deal with ionic composition and its interaction with major organic acids such as malic, citric or fumaric acids (Corol et al. 2014; Deborde et al. 2017; Fan et al. 1997; Kruger et al. 2008). Moreover, for high-throughput fingerprinting, extraction can be performed directly with deuterated solvents like ethanol-*d6* or methanol-*d4*. Although the former is less toxic, it is currently more expensive than methanol-*d4*, which is used widely.

Plant sample specificities and associated NMR profiling protocols have been previously described (Allwood et al. 2011; Mannina et al. 2012; Schripsema 2009). ^1^H-NMR profiling is usually considered as more robust than LC-MS profiling based on repeatability (Moing et al. 2004; t’Kindt et al. 2009). However, there is emerging interest in improving 1D ^1^H-NMR repeatability and throughput to analyze larger sample sets composed of the same tissue type or of different kinds of tissue extracts (Corol et al. 2014; Fan et al. 1997; Tredwell et al. 2016), and the possible need for multi-lab acquisitions for very large sample sets. In this tutorial, we propose precautions and actions to improve NMR repeatability when profiling plant sample series. The steps involved are standardization of extract preparation, preparation of the NMR instrument, verification of sample spectra quality and spectra processing, with partial automation of dedicated steps. As a proof of concept, a profiling experiment on wheat samples with spectra acquired at three different sites on instruments of different field strengths and manufacturers is presented.

## Standardizing the preparation of extracts and NMR instrument before spectra acquisition

To improve spectra quality and the reproducibility of plant extracts, the preparation of extracts, the preparation of NMR instrument and shimming can be standardized as described below.

### Preparation of semi-polar extracts

The critical steps of extract preparation are described below and more details can be found in Online Resource 1. A quantity of 20 to 100 mg of fine powder (best grinding quality with particle size of 70 to 150 µm or 200 to 100 mesh) is recommended. Tests must be performed to optimize extract concentration by checking for the linear response of exploitable spectral information (signal-over-noise ratio, S/N ratio) to powder quantity, but also for spectral quality. A compromise must be found: on one hand, one should not dilute too much because extracts with low concentrations are easier to shim but provide a low number of resonances detected; on the other hand, one should not concentrate too much extracts because concentrated extracts are more difficult to shim with broader resonances due to higher viscosity (Halabalaky et al. 2014). The extraction solution is prepared with two deuterated solutions. The first solution contains D_2_O, phosphate salts and ethylene diamine tetraacetic acid sodium salt (EDTA). Its pH is adjusted to a value of 6 (pH_apparent_) using deuterated solutions (NaOD and DCl). The second solution contains MeOD-*d4*. The quantity of residual signals depends on the quality of the chemical products used. Deuterated products are better even though they are more expensive. However, a compromise is to perform extraction with D_2_O and MeOD-*d4* only, and non-deuterated phosphate salt and EDTA. If non-deuterated EDTA is used, resonances of free EDTA (singlets at 3.72 and 3.42 ppm) or EDTA complexed with Ca^2+^ or Mg^2+^ (broad signals at 3.2 and 2.7 ppm) are observed (Asiago et al. 2008; Corol et al. 2014; Han et al. 2007).

The first extraction step consists in adding MeOD-*d4* to the plant powder contained in a polypropylene microtube under a hood. If an automated dispenser is used, the viscosity of methanol requires the optimization of some parameters such as aspiration speed, ejection speed and special tubing. If the procedure is manual, a positive displacement pipette is recommended to distribute the accurate volume. The deuterated buffer solution is then added. This solution must be stored carefully. Solutions must be added accurately and reproducibly. A homogenization step (usually 5 min) and a sonication step (30 min at 30 °C) are then needed. When plant samples containing phytopathogenic microorganisms are being tested, an additional thermal inactivation step is recommended (90 °C for 2 min, (Ward et al. 2010); see Online Resource 2 for illustration of probable residual enzyme activity in NMR tube after extraction of a fungi-infected wheat sample without heating). A centrifugation step is needed to remove solid parts (classically 10 min at 4 °C, 15,000 or 20,000×*g*). Its temperature can be optimized to remove most polysaccharides and proteins. A defined volume of supernatant is transferred to another plastic vial and its pH_apparent_ has to be carefully adjusted to 6 (tolerance 0.02) using NaOD or DCl solutions. Robotized pH adjustment is a good alternative to manual adjustment. The amount of 3-trimethylsilylpropanoic-*2,2,3,3-d4* acid sodium salt (TMSP) for chemical shift calibration should be adjusted according to the sample type. The pH-adjusted extracts are then transferred to NMR tubes that must be of uniform and suitable quality: economy disposable tubes are not recommended. If high-quality tubes are re-used, the tube cleaning and drying steps are crucial and should be checked. A constant volume must be transferred to the NMR tube (e.g. 600 µl for a 5 mm tube). At least one blank extraction should be performed in the preparation workflow, consisting in running extraction without plant powder.

### Standardized preparation of NMR instrument

To prepare the NMR instrument before profiling of a sample series, three types of NMR tubes are needed: a calibration sample of MeOD-*d4* for temperature calibration, and a sucrose test-tube and a blank-extract tube for optimization of shim matrices. First, it is recommended to check and calibrate the temperature of the NMR spectrometer by means of a calibration sample of MeOD-*d4* prepared and measured with the SOP proposed previously (Findeisen et al. 2007). In addition, a stable temperature in the NMR probe will prevent variations of chemical shifts, especially the water peak for water suppression. We recommend checking the stability of temperature over time in the NMR probe (e.g. with the “edte” module for Bruker, and in the temperature monitor for JEOL). NMR spectra are usually acquired at a temperature of 25 or 27 °C, or the same given temperature for complex extracts and pure compounds to facilitate spectra annotation and quantification. Indeed, the effects of uncontrolled temperature on chemical shifts and quantification have already been discussed elsewhere (Emwas et al. 2015). To ensure that acquisition is performed at the right temperature, a waiting period of 5 min in the magnet is recommended for temperature equilibration before spectra acquisition.

Even if NMR spectrometers are certified annually, we recommend running a 3D shim map (Sukumar et al. 1997) before NMR acquisition campaign with a commercial test-tube containing 2 mM sucrose added with 0.5 mM DSS, 2 mM NaN_3_ in 90–10% (v/v) H_2_O-D_2_O, generally used to calibrate water suppression and spectrometer sensitivity. Shim matrices are then optimized on the blank-extract tube and the spectral quality of this extract must be identical to that of the test-tube used for the specifications of the device (full width at half maximum (FWHM) of TMSP < 1 Hz or FWHM for the residual signal of methanol < 1.35 Hz with LB 0.3 Hz) see Online Resource 3. Finally, tubes of a few selected but representative sample extracts are to select 1D pulse sequences with presaturation (“noesypr1d” or “zgpr” for Bruker and “proton with presaturation” or “noesy_abs” for JEOL) (Giraudeau et al. 2015). The residual water signal can be eliminated in several ways (Giraudeau et al. 2015). In the case of freeze-dried samples, a classic water suppression pulse sequence is adequate. The systematic application of the same acquisition parameters (e.g. spectral window and receiver gain) is recommended.

## Steps that benefit from standardizing the preparation of samples and instrument

Depending on the ionic composition of plant samples, which is difficult to anticipate, two parameters which may induce variations of chemical shifts should be controlled along with temperature, namely sample pH and chelation of paramagnetic ions.

### pH adjustment

Slight differences of pH among sample extracts can induce variation of some chemical shifts if uncontrolled, pH adjustment of each sample extract may limit these variations and simplify post-acquisition processing including peak alignment. Although the extraction solution contains 45 mM phosphate buffer salts, this is not always adequate to deal with acidity of plant samples, especially immature fruit tissues. As increasing its concentration leads to salt precipitation in the methanol water extraction solution, pH adjustment is compulsory. See Online Resource 4 for pH variability of representative plant extracts.

### Limitation of paramagnetic ions effects

**Fig. 2:**
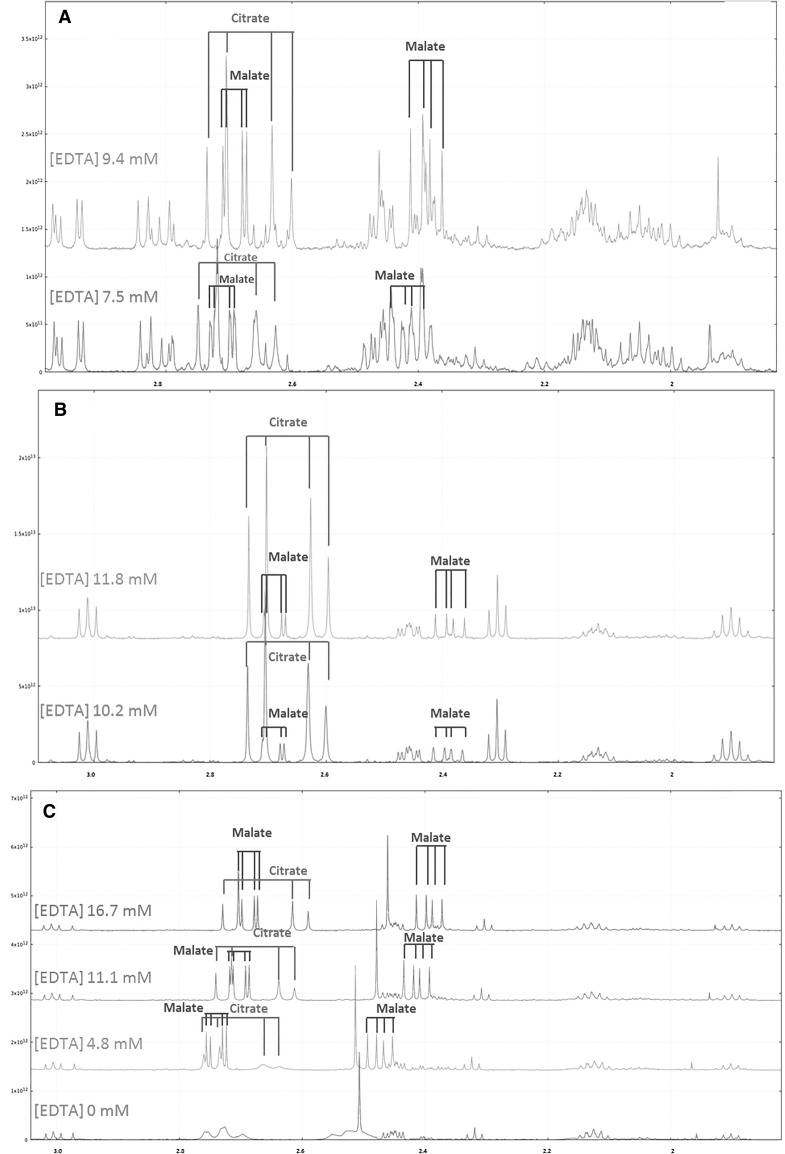
Effect of EDTA addition on ^1^H-NMR spectra resonances in the 3.1–1.8 ppm region for plant semi-polar methanolic extracts. **A** Wheat spikelet extract (500 MHz Bruker). **B** Tomato fruit pericarp extract (500 MHz Bruker). **C** Preliminary test without pH control to determine EDTA concentration in test tube of flax root extract (600 MHz Bruker)

Plant tissues or exudates often contain paramagnetic ions such as Cu^2+^, Fe^2+^ and Fe^3+^, Mn^2+^ (Deborde et al. 2017; Fan et al. 1997). Carboxyl and hydroxyl groups of organic acids such as malate and citrate interact with these ions to form chelates. Magnetic properties of hydrogen nuclei of organic acid methylene groups of these chelates are modified and turn to be invisible by ^1^H-NMR. Two options are used to limit the paramagnetic ions effects, either pass the sample extract through a cation-exchange resin column (Corol et al. 2014; Fan et al. 1997; Moing et al. 2004) or add EDTA (Allwood et al. 2011; Corol et al. 2014) to the sample. In the latter case, EDTA can be added during and/or after the extraction step. Figure 2 shows the impact of EDTA-*d12* addition on proton spectra of wheat spikelet (Fig. 2A), tomato fruit (Fig. 2B) and flax root (Fig. 2C) extracts. For wheat spikelet and tomato fruit extracts, EDTA concentration was adjusted after acquisition of selected samples and additional quantities of EDTA (32 µl and 64 µl of 98 mM EDTA solution, respectively) were added to reach a concentration of about 10 mM, see Online Resource 4.

### Shimming and acquisition parameters

Whenever possible, the data acquisition step should be done immediately after NMR tube preparation. The storage of extracts in a freezer is not ideal as possible precipitation of compounds may happen. The choice of the acquisition temperature is based on a compromise between possible degradation and precipitation of compounds. NMR tubes are placed in the sample handler every 24 h (stability assumed at room temperature, see Online Resource 2 for stability check). The order of tubes placement is important (Defernez and Colquhoun 2003). A randomized order is advised for large sample-sets, but in the case of small series it may not produce the expected effect. It may be better to alternate the tubes of the different biological conditions. To standardize acquisition, it is recommended to include an automation routine in the acquisition pipeline which includes temperature homogenization, automatic probe tuning and matching, locking solvent, shimming, and 90° calibration pulse determination as detailed in Online Resource 3. A long relaxation delay is advisable. In our conditions, many metabolites have a T1 less than 4 s. We chose 20 s but this value must be optimized according to the specificities and objectives of the study.

## 1D ^1^H spectra quality criteria

**Fig. 3:**
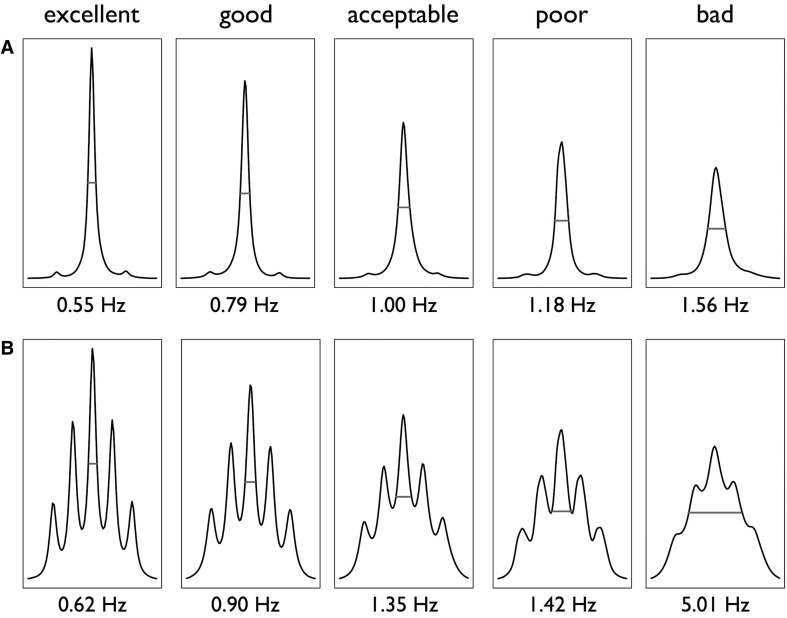
Impact of shim quality on full width at half maximum (FWHM) of TMSP singlet (**A**) or of methanol deuterated (CD_2_HOH) multiplet (**B**), in *Arabidopsis* semi-polar methanolic extracts. Bruker 600 MHz spectra recorded at 27 °C with a 0.3 Hz line broadening for exponential window multiplication of FID. FWHM measurement is represented by red segment

As soon as possible after spectra acquisition, checking for spectra quality is a critical step to re-record or remove sample spectra that do not meet quality criteria. Therefore, the quality of all sample spectra should be evaluated before spectral data reduction or peak integration for data mining. It can be evaluated using the full width at half maximum (FWHM) that can be calculated using routines such as described in Online Resource 5 and the coefficients of variation (CV) of the signal-to-noise (S/N) ratio of selected peaks. If TMSP has been added as a spectral chemical shift reference in the extract, this compound can be used to assess the quality of the spectrum. If such a chemical shift reference has not been added (for example, if TMSP complexes with compounds of the sample extract), the solvent signal can be used to assess it. In both cases, the residual methanol (CHD_2_OD) signal can be used. As shown in Fig. 3, a FWHM of 0.55 Hz and of 0.62 Hz for TMSP and methanol respectively, with an LB of 0.3 at a temperature of 27 °C, gives an excellent quality of signal. Indeed, a very good resolution is necessary in metabolomics to discriminate the different signals, especially in areas with spectral overlap. The coefficients of variation of FWHM and of the S/N ratio are also important to ensure that the acquisition quality during the experiment remains stable. As in a metabolomic study, groups of spectra are compared, the spectral quality must remain constant. It is important to check that the S/N ratio remains relatively uniform across the different levels of the biological factors in the experiment, and that its CV remains lower than 10%. In any case, it is recommended to aim at an FWHM lower than 1 Hz for TMSP and 1.35 Hz for the residual methanol (CHD_2_OD) signal, with a CV lower than 10% for FWHM and S/N.

When a sample does not meet the spectra quality criteria, possibly because the tube was not introduced correctly into the cryo-magnet, it is still possible to re-run an acquisition which may improve shimming. Knowledge of the sample extracts is also helpful because depending on the sample, the shimming step is more or less easy. For example, Table 1 shows that depending on organ type or harvest time, the shim quality and the CV of the FWHM of TMSP or of methanol may vary. In experiment 1, the leaves and the youngest stage had a less homogeneous shim (larger CV) than the older harvest stages. In Table 1 of experiment 2, FWHM was lower than 0.59 Hz with a CV lower than 4% for TMSP, and FWHM was lower than 0.65 Hz with a CV lower than 5% for the residual methanol signal. In experiment 2 in which the number of extracts was lower than in experiment 1 and the type of sample differed, the reproducibility was greater. In Table 2, whatever the field strength, the mean FWHM was lower than 0.85 Hz for TMSP and 1.02 Hz for the residual methanol signal with a CV lower than 11% for the same sample extracts of wheat recorded on the three spectrometers. The CV of S/N ratio was lower than 8% in all cases, indicating a good reproducibility between samples. The quality criteria requirements have therefore to be adapted according to the number (stability of the NMR device during manipulation) and the type of samples studied (plant extracts more or less concentrated, and stability of the magnetic field more or less easy to obtain). Therefore, knowledge about the biological extract greatly helps when estimating the spectral quality that can be achieved. All the sample extract spectra fulfilling the spectra quality criteria can then be processed for alignment and bucketing or peak integration.

**Table 1:** Example of parameters obtained for checking shim quality with a large-size plant dataset comprising more than 450 samples of greenhouse-grown flax (Experiment 1) and a medium-size plant dataset comprising about 90 samples of greenhouse-grown *Arabidopsis* (Experiment 2)

Sample type	TMSP signal			CHD_2_OD signal			Number of NMR spectra
	FWHM (Hz)		S/N	FWHM (Hz)		S/N	
	Mean	CV (%)	CV (%)	Mean	CV (%)	CV (%)	
Experiment 1							
Flax							
All	0.65	9	8	0.73	10	9	468
Roots	0.66	8	8	0.73	9	9	156
Leaves	0.65	10	8	0.73	12	9	155
Stem	0.65	9	7	0.72	9	6	157
7 DAS	0.66	12	9	0.74	14	8	108
10 DAS	0.65	9	8	0.72	10	9	120
15 DAS	0.65	7	7	0.73	8	8	120
20 DAS	0.64	7	7	0.72	8	7	120
Experiment 2							
*Arabidopsis*							
All	0.59	3	4	0.65	3	5	92
WT	0.59	2	4	0.65	2	5	46
M	0.59	3	4	0.65	4	5	46
Roots	0.59	3	4	0.65	3	5	44
Leaves	0.58	2	4	0.64	2	4	48

Values (mean values and coefficients of variation, CV) obtained for full width at half maximum (FWHM) and signal-to-noise ratio (S/N) calculations of TMSP singlet and CHD_2_OD multiplet for methanolic extracts. First row of the table shows all data used for calculations in each experiment. Experiment 1: other rows of the table illustrate the effect of two biological factors, harvested organ (roots, leaves and stem) and cultivation duration (7 days after sowing (DAS), 10 DAS, 15 DAS, and 20 DAS for entire plant), on these parameters. Experiment 2: other rows of the table illustrate the effect of plant genotype, wild type (WT) or mutant (M, a mutant of the phenylpropanoid pathway), and harvested organ (roots or leaves) on these parameters. Data were obtained on Bruker 600 MHz spectra recorded at 27 °C with a noesypr1d pulse sequence and a 0.3 Hz line broadening for exponential window multiplication of FID. Spectra obtained from the doctoral theses of Sylvain Lecomte (Lecomte 2017) or Cédric Decourtil (Decourtil 2016)

**Table 2:** Influence of NMR field frequency on TMSP and CHD_2_OD signal quality in a small-size plant dataset of 22 wheat samples

Field strength (MHz)	TMSP signal			CHD_2_OD signal			Number of NMR spectra
	FWHM (Hz)		S/N	FWHM (Hz)		S/N	
	Mean	CV (%)	CV (%)	Mean	CV (%)	CV (%)	
400	0.85	8	7	1.04	11	5	22
500	0.74	11	8	0.78	8	4	22
600	0.61	6	6	0.67	6	4	22

Values (mean values and coefficients of variation, CV) obtained for full width at half maximum height (FWHM) and signal-to-noise ratio (S/N) calculations of TMSP singlet and (CHD_2_OD) multiplet for methanolic extracts of greenhouse-grown wheat spikelets

Data were obtained on JEOL 400 MHz, Bruker 500 MHz or Bruker 600 MHz spectrometers, and spectra were recorded at 27 °C with 1D ^1^H presaturation pulse sequence and a 0.3 Hz line broadening for exponential window multiplication of FID

## Spectra processing

Spectra processing consists in extracting information from NMR spectral data that can be used to address a biological issue. In this tutorial, we consider untargeted metabolic fingerprinting (Krishnan et al. 2005) which does not focus on a particular set of metabolites. The first step known as “spectra pre-processing” consists in transforming raw spectra in the time domain to spectra in the frequency domain (i.e. apodization and zero filling of the Free Induction Decay (FID) followed by a Fourier Transformation). The phase is then corrected to obtain an absorption line shape. Subsequently, the “spectra processing” step, which is an intermediate step between raw spectra and data analysis, is performed in the frequency domain. It consists in preserving as much as possible the variance relative to the chemical compound signals contained in the NMR spectra, while reducing other types of variance induced by different sources of biases such as baseline, noise or peak misalignment. Because an NMR spectrum may contain several thousand points, and therefore variables, data reduction or bucketing is commonly used to reduce data dimensionality. In bucketing, the spectra are divided into spectral regions or buckets (also called bins) and the total area within each bucket is calculated to represent the original spectrum. Finally, to make all spectra comparable with each other, variations in the overall concentrations of samples must be accounted for. In NMR plant metabolomics, since the samples are generally lyophilized and then cryo-grinded, total intensity normalization is often used so that all spectra correspond to the same overall concentration, provided the solvent spectral region is properly excluded from the bucket integration zones. Normalization with the quantity of extracted powder is another possibility. Detailed explanations can be obtained in a recent review (Deborde et al. 2017).

For the examples presented in the present tutorial, all the spectra processing steps mentioned above were performed using the NMRProcFlow web application (Jacob et al. 2017) that provides a complete set of tools for processing and visualizing 1D NMR data recorded on Bruker, JEOL or Varian/Agilent spectrometers, within an interactive interface based on spectra visualization. In addition, it allows users to record and replay their spectra processing workflow, ensuring reproducibility. Automatic processing can thus be considered on sets of spectra acquired under similar conditions. Details about spectra processing are given in Online Resource 5. Indeed, automation ensures reproducibility and traceability, and guarantees that all spectra are processed in the same way according to a macro-command list defined and checked by an NMR expert.

## Robustness and reproducibility

To check the robustness and reproducibility of all the standardization steps described above, we used a collection of 22 samples of wheat spikelet extracts (with two biological groups, 5 and 14 days after flowering, DAF, as described in Online Resource 1) with spectra collected at three different sites on instruments of different field strengths and manufacturers following the recommendations described above.

As a first check, several NMR spectra were visualized in the frequency domain before processing. The NMR spectra were chosen so that they corresponded to the same biological sample taken from the spectra set acquired on the three instruments. The stacked visualization of spectra of a representative sample (Online Resource 6) shows a global good reproducibility between instruments: most of the signals are present on all spectra obtained with the various instruments and with similar intensities. Only a few clusters of peaks seem more or less resolved according to the magnetic field strength and thus the resolution capacity of the spectrometers, as expected.

**Fig. 4:**
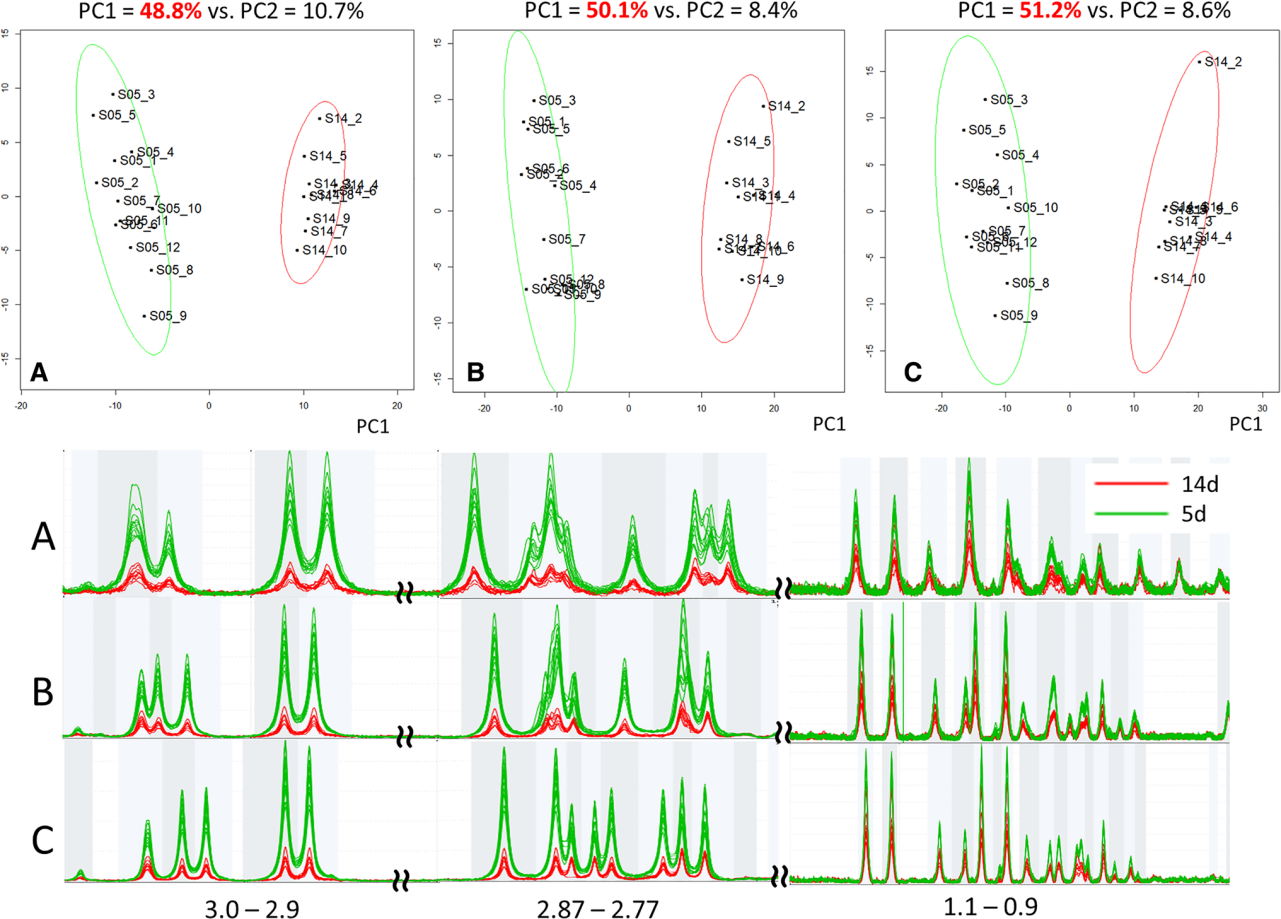
PCA for each ^1^H-NMR dataset of 22 wheat samples acquired on a given NMR instrument and zoom in on a spectral region. *Top*: Scores plot of PCA for semi-polar methanolic extracts of spikelets at 5 DAF (green ellipse) or 14 DAF (red ellipse), for 400 MHz JEOL instrument (**A**, 224 buckets), 500 MHz Bruker instrument (**B**, 397 buckets), 600 MHz Bruker instrument (**C**, 465 buckets). Ellipses represent 95% of confidence level. Bottom: Comparison of several spectral regions (3–2.9, 2.87–2.77 and 1.1–0.9 ppm) for 5 (green) and 14 (red) DAF for 400, 500 and 600 MHz spectrometers with bucket positions for the entire dataset

Then, we processed each of the three sets of spectra as identically as possible as described in Online Resource 5. The intelligent bucketing mode (De Meyer et al. 2008) in NMRProcFlow was chosen, so that each bucket exactly matched one resonance peak. Consequently, the buckets have a chemical meaning, since the resonance peaks are the fingerprints of chemical compounds. This procedure makes it possible to compare not only the global variance between the two stages (5 and 14 DAF), but also each resonance separately (especially their ratios between experimental conditions) across the entire spectra. Regarding the global variance, a principal component analysis (PCA) was applied to each data matrix resulting from the intelligent bucketing step for a set of spectra. The PCA scores (Fig. 4, *Top*) show not only the same discrimination pattern of the two biological groups (5 and 14 DAF) for Bruker 500 MHz, 600 MHz and JEOL 400 MHz spectrometers, but also a very close explained variance for the main component (principal component 1, PC1). It is noteworthy that each sample is located almost in the same region on each of the PCA scores plots. This testifies to the good repeatability between several instruments from different manufacturers and with different characteristics, as well as good spectral processing robustness. Regarding the bucket variabilities, the distributions of bucket ratios between 5 and 14 DAF means exhibited a very similar standard deviation for the three datasets (Online Resource 5). We compared the positions of the buckets produced by intelligent bucketing for the 400, 500 and 600 MHz datasets (Fig. 4 *Bottom*, Online Resource 7). As expected the bucket boundaries depend on the instrument resolution. Because the coupling constants are field-independent, overlapping between resonance peaks may occur at lower magnetic field strength. However, despite the difference in instrument resolution, the resonance peaks on each spectra set were well detected and determined by the bucketing process, preserving the chemical patterns since the intelligent buckets matched the fingerprints of the chemical compounds. To pave the way for detailed biological interpretation of PCA loadings, which is not the purpose of this tutorial, (Online Resource 7), we performed a clustering analysis based on a threshold applied on the bucket correlation matrix (Jacob et al. 2013). This demonstrated that for the three spectra sets (i) the overall structure of the PCA loadings was very similar regarding the bucket-cluster position on each loadings plot, the cluster number depending on the instrument resolution, and (ii) that after a preliminary automatic matching (Jacob et al. 2013) of bucket clusters with reference NMR spectra libraries, we found a set of the same metabolites associated with bucket clusters located in the same PCA loadings plot regions (Online Resource 7).

## Conclusions

In this tutorial, we have focused upon standardization of plant extract preparation, preparation of NMR instruments, verification of sample spectra quality and spectra processing with partial automation of dedicated steps. Automation routines for spectrometer programming are also provided. The necessity to control paramagnetic ion concentrations in plant extract preparations was underlined, especially for key metabolites such as malic and citric acids, for studying primary plant metabolism by NMR-based metabolomics. However, it is important to stress that there is no universal EDTA concentration established for all plant tissues and that optimal EDTA concentrations should be determined experimentally for each specific plant tissue from an organ at a given developmental stage.

To our knowledge, this is the first plant metabolomic inter-laboratory test with spectrometers from two different manufacturers. As a proof of concept, a profiling experiment on wheat samples with spectra acquired at three different sites on instruments of three different field strengths and manufacturers provided very similar chemical and biological information. Best practice recommendations, including standardization and partial automation (rarely detailed in the method part of biological articles), regarding the NMR analysis of samples for NMR-based metabolomics that are partly common and specific to the type of samples, and recommendations have been published recently for human urine samples (Emwas et al. 2015). Besides software tools such as those used here for the proof of concept example on plant samples and those described by Eghbalnia et al. (2017), databases are also needed to improve the rigor, robustness, reproducibility, and validation of metabolomics studies (Eghbalnia et al. 2017).

Two types of information are researched during metabolomic fingerprinting analysis: spectral region integrations as detailed here, and then identification of discriminant spectral regions playing a role in the biological process under study. Therefore, after a “fingerprinting” analysis, simple 2D experiments like ^1^H 2D J-resolved can be performed (Ludwig and Viant 2010) when strong signal overlaps are suspected. For more robust identification (Everett 2015), other 2D experiments (COSY, HSQC, HMBC) are also often performed on representative samples. Interest in the standardization and semi-automation of 1D and 2D NMR spectra annotation is currently growing as reported for mass spectrometry, (Blaženović et al. 2018), to obtain faster and more rigorous interpretation of NMR-based metabolomic fingerprints and profiles.

## Electronic supplementary material

Below is the link to the electronic supplementary material.

Supplementary material 1 (PDF 184 KB)

Supplementary material 2 (PDF 246 KB)

Supplementary material 3 (PDF 269 KB)

Supplementary material 4 (PDF 329 KB)

Supplementary material 5 (PDF 701 KB)

Supplementary material 6 (PDF 435 KB)

Supplementary material 7 (PDF 956 KB)

## Data Availability

The metabolomics and metadata of the wheat spikelet experiment reported in this paper are available via Portail Data Inra (https://data.inra.fr) study identifier ERCVZR (10.15454/ERCVZR).

## Abbreviations

DAF: Days after flowering
DAS: Days after sowing
DSS: 4,4-Dimethyl-4-silapentane-1-sulfonic acid
EDTA: Ethylene diamine tetraacetic acid
FID: Free induction decay
FWHM: Full width at half maximum
NaN_3_: Sodium azide
NMR: Nuclear magnetic resonance spectroscopy
PCA: Principal component analysis
S/N: Signal-to-noise
TMSP: 3-Trimethylsilylpropanoic-*2,2,3,3-d4* acid sodium salt
