# Supplementary material for: Optimizing 1D 1H-NMR profiling of plant samples for high throughput analysis: extract preparation, standardization, automation and spectra processing
Source: Metabolomics. 2019 Feb 26;15(3):28. doi: 10.1007/s11306-019-1488-3 (PMC6394467; doi:10.1007/s11306-019-1488-3)
Supplement: Supplementary file 1 — Supplementary material 1 (PDF 184 KB) [file 11306_2019_1488_MOESM1_ESM.pdf]

Journal: Metabolomics
Title: Optimizing 1D 1H-NMR profiling of plant samples for high throughput analysis: extract preparation, standardization, automation and spectra processing
Authors: Catherine Deborde, Jean-Xavier Fontaine, Daniel Jacob, Adolfo Botana, Valérie Nicaise, Florence Richard-Forget, Sylvain Lecomte, Cédric Decourtil, Kamar Hamade, François Mesnard, Annick Moing, Roland Molinié

Online resource 1. Plant culture and sample preparation protocol for the wheat spikelet NMR experiment

Plant culture conditions and sample harvest

Triticum durum (durum wheat) *cv.* Sculptur plants were cultivated in a greenhouse. Spikelet without awns were harvested at 5 or 14 days after flowering (DAF), frozen in liquid nitrogen and stored at -80°C before freeze-drying. To evaluate the impact of a pathogen on the stability of plant methanolic extracts, some wheat kernels were inoculated with Fusarium, a plant pathogenic fungus, at the flowering stage. A biological replicate was constituted of 5 spikelets at 5 DAF and 2 spikelets at 14 DAF.

Sample preparation and extraction

Sample weight: 40 ± 1 mg DW in 2 ml polypropylene microtube with conical bottom with skirt (e.g. 2.0 ml SC Micro Tube protein LB from Sarstedt).

Chemical products and extraction solutions:

- D_2O 99.9%D
- NaOD (99.5% D) 40% w/w
- DCl (99.8% D) 11.8 N
- 1 M NaOD in D_2O with 0.01% w/v NaN_3.
- 1 M DCl in D_2O with 0.01% w/v NaN_3.
- 58 mM TMSP (3-trimethylsilyl propionic-*2,2,3,3-d4* acid, sodium salt, 98%D) in D_2O.
- MeOD-*d4* 99.8%D, (HOD + D_2O) < 0.03%
- 98 mM EDTA-*d12* in D_2O at $pH_{apparent}$ 8.7. Dissolution of acidic form of EDTA-*d12* in D_2O with drops of NaOD 1 M solution to $pH_{apparent}$ 8.7. Solution kept at 4°C for 1-2 months
- 90 mM phosphate ($KD_2/K_2D\ PO_4$ in D_2O) buffer solution at $pH_{apparent}$ 6, containing 11 mM EDTA-*d12*

Extraction and pH adjustment

Manual Extraction: add 750 µl MeOD-*d4* (with positive displacement pipette) under a hood (with gloves, glasses and protective clothes). Vortexing for 1 min at maximum speed. Then add 750 µl of buffer solution at $pH_{apparent}$ 6 (phosphate buffer $KD_2/K_2D\ PO_4$ 90 mM, 11 mM EDTA-*d12*). Vortexing for 1 min at maximum speed.

Automated Extraction: add with a robot 1,500 µl of a solution at $pH_{apparent}$ 6.0 (50/50, v/v MeOD-*d4* / phosphate buffer $KD_2/K_2D\ PO_4$ 90 mM, 11 mM EDTA-*d12*) under a hood (with gloves, glasses and protective clothes). Vortexing for 1 min at maximum speed.

Ultrasonic bath for 10 min, vortexing for 1 min at maximum speed.

Ultrasonic bath 10 min, vortexing for 1 min.
Ultrasonic bath for 10 min, vortexing for 1 min.

Adaptation: Ultrasonic bath can be run on ice if thermosensible specialized metabolites are expected in the samples.

Centrifugation: 15,493 *g* (13,000 rpm) 4°C for 10 min for macromolecular compound precipitation. Collect 900 µl of supernatant with positive displacement pipette and dispense it into a 2 mL vial compatible with pH adjustment step. In case of floating particles at the air-liquid interface for some plant samples, use a Pasteur pipette to withdraw the maximum of supernatant volume (at least 600 µl, the lower limit required for pH adjustment step) or program carefully the withdraw needle depth of an automated dispenser to cross the interface and not withdraw particles.

Adjust $pH_{apparent}$ to 6.00 ± 0.02 with NaOD 1 M and/or DCl 1 M solution manually or by means of BTpH titration robot (Bruker, Karlsruhe, Germany).

Store at 4°C for a night if not analyzed immediately, or at -20°C in sealed bag with desiccant. If storage, before opening the vials, let them warm up at room temperature in a desiccator. If needed, centrifuge vials. To a 5-mm NMR tube (e.g. 5 mm 507-pp-7 Wilmad) add 8 µl of TMSP solution and dispense 800 µl of extract. Adjust volume of TMSP solution to extract volume.

At the end of the standardized preparation of NMR instrument and preliminary test on a few selected but representative sample extracts for the selection of 1D pulse sequences with presaturation, the resolution of citrate and malate resonance pattern is checked and if needed an additional EDTA-*d12* solution volume may be added to each NMR tube of sample set (see Online resource 4).
