# Supplementary material for: Optimizing 1D 1H-NMR profiling of plant samples for high throughput analysis: extract preparation, standardization, automation and spectra processing
Source: Metabolomics. 2019 Feb 26;15(3):28. doi: 10.1007/s11306-019-1488-3 (PMC6394467; doi:10.1007/s11306-019-1488-3)
Supplement: Supplementary file 2 — Supplementary material 2 (PDF 246 KB) [file 11306_2019_1488_MOESM2_ESM.pdf]

Journal: Metabolomics
Title: Optimizing 1D 1H-NMR profiling of plant samples for high throughput analysis: extract preparation, standardization, automation and spectra processing
Authors: Catherine Deborde, Jean-Xavier Fontaine, Daniel Jacob, Adolfo Botana, Valérie Nicaise, Florence Richard-Forget, Sylvain Lecomte, Cédric Decourtil, Kamar Hamade, François Mesnard, Annick Moing, Roland Molinié

Online resource 2. Example of extract stability study for wheat samples

Illustration of the effect of partial instability of semi-polar extract in NMR tube after methanolic extraction of wheat samples without heating for *Fusarium* non-infected and *Fusarium* infected samples. A. 1D ^{1}H spectra (500 MHz Bruker) portions of wheat spikelet at 5 DAF at 1 day (red), 2 days (green) and 37 weeks (blue) after extraction and storage at 4°C. B. Effect of storage on the quantity of sucrose, glucose and alanine normalized to TMSP intensity, A.U. arbitrary unit. The non-infected extract is stable, whereas the *Fusarium* infected one shows a conversion of sucrose into glucose probably due to a residual enzyme activity (Akinrefon 1968, deb Dutta et al. 2018).

A

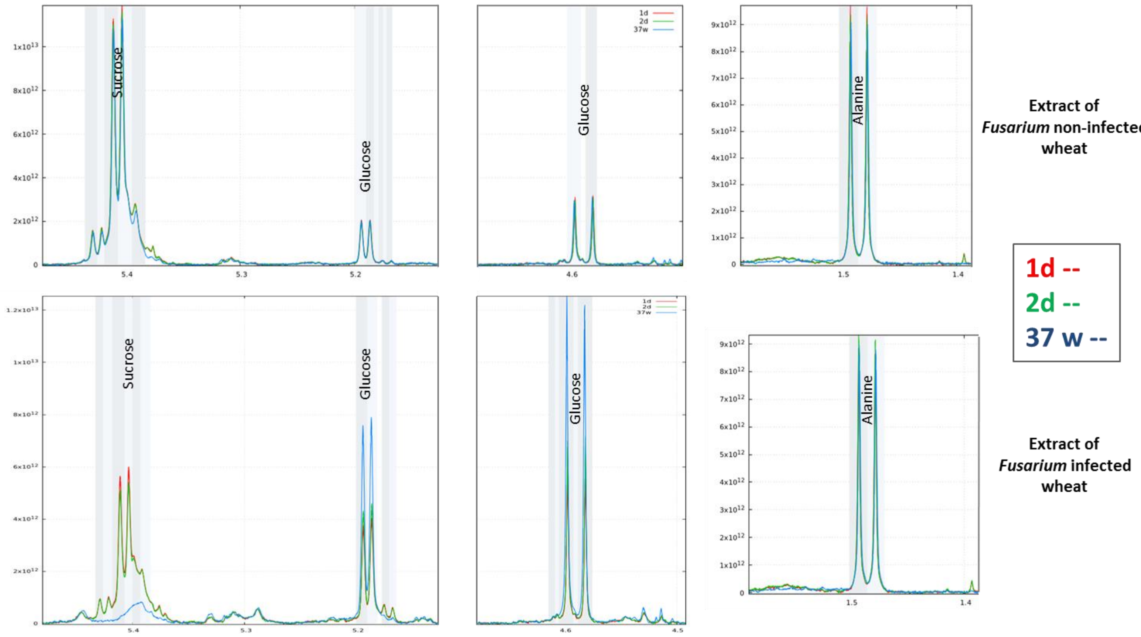

B

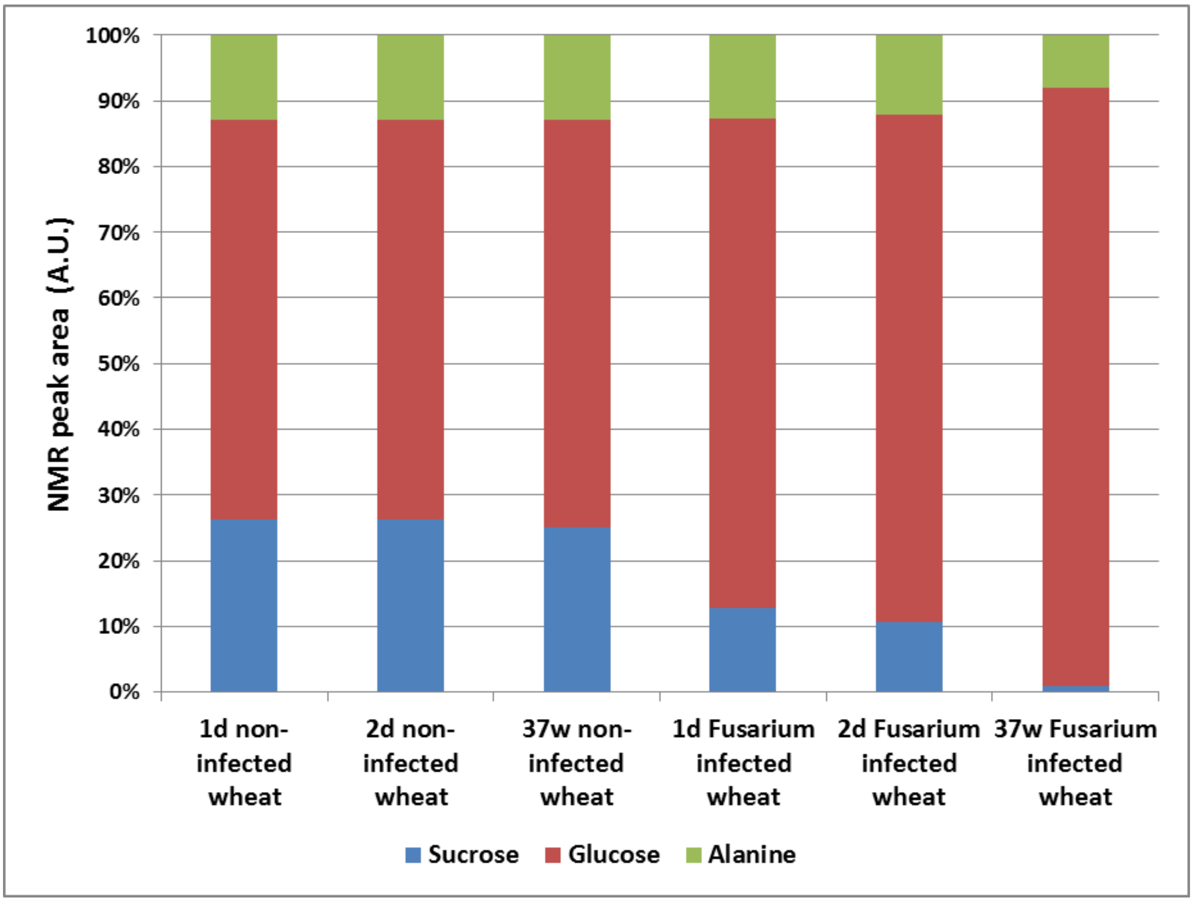

Akinrefon, O. A. (1968). Studies on the α-L-arabinofuranosidase of *Phytophthora palmivora* (Butl.) Butl. . *New Phytologist, 67*(3), 543-556, doi:10.1111/j.1469-8137.1968.tb05482.x.

deb Dutta, S., Tarafder, M., Islam, R., & Datta, B. (2018). Characterization of cellulolytic enzymes of *Fusarium* soil Isolates. *Biocatalysis and Agricultural Biotechnology, 14*, 279-285, doi:10.1016/j.bcab.2018.03.011.
