# Supplementary material for: Optimizing 1D 1H-NMR profiling of plant samples for high throughput analysis: extract preparation, standardization, automation and spectra processing
Source: Metabolomics. 2019 Feb 26;15(3):28. doi: 10.1007/s11306-019-1488-3 (PMC6394467; doi:10.1007/s11306-019-1488-3)
Supplement: Supplementary file 3 — Supplementary material 3 (PDF 269 KB) [file 11306_2019_1488_MOESM3_ESM.pdf]

Journal: Metabolomics
Title: Optimizing 1D ^{1}H-NMR profiling of plant samples for high throughput analysis: extract preparation, standardization, automation and spectra processing
Authors: Catherine Deborde, Jean-Xavier Fontaine, Daniel Jacob, Adolfo Botana, Valérie Nicaise, Florence Richard-Forget, Sylvain Lecomte, Cédric Decourtil, Kamar Hamade, François Mesnard, Annick Moing, Roland Molinié

Online resource 3. NMR spectra acquisition methods

3.1 400 MHz NMR JEOL instrument

NMR spectra were recorded on a 400 MHz ECZ-S JEOL (Tokyo, Japan) spectrometer equipped with a double resonance ROYAL probe (5 mm NMR tube) and an autosampler (ASC-30) that was used at room temperature.
Routines were managed by jaf scripts available upon request to A. Botana (JEOL UK, adolfo.botana@jeoluk.com).
^{1}H-NMR spectra were obtained using Delta 5.3 software. Sample temperature was regulated at 27°C, and once the temperature reached 300 K there was a delay of 2 minutes for temperature stabilization. Automatic tuning and matching of the probe was carried out for each sample. Samples were not spun. Standard shim settings from a saved file were loaded with each new sample. MeOD signal (from the extraction solution) was used for the field/frequency lock and shims were optimized for each sample using gradient shimming (Z1 to Z6) followed by Fast Autoshim of (X, Y, XZ and YZ) shims. The 90° pulse length was determined for each sample using the Auto_1H_90_Degree routine. Instrument specific acquisition parameters are summarized in the **Table S3.1**.

3.2 500 and 600 MHz NMR Bruker instruments

NMR spectra were recorded on a Bruker Avance III 600 MHz spectrometer certified every year by Bruker (Wissembourg, France). The spectrometer operated at 600.13 MHz for ^{1}H, using a three nuclei (^{1}H, ^{13}C, ^{15}N) inverse ATMA head TXI 5 mm z-gradient probe. It was equipped with an autosampler (SampleXpress) that was used at room temperature.

NMR spectra were recorded on a Bruker Avance III 500 MHz spectrometer (Wissembourg, France). The spectrometer operated at 500.13 MHz for ^{1}H, using an inverse ATMA BBI 5 mm z-gradient probe. It was equipped with an autosampler (BACS-120) that was used at room temperature.

The TopSpin 3.2 (Bruker, Karlsrühe, Germany) software was used and the IconNMR software module controlled the automation. Sample temperature was regulated at 27°C with an interval of approximately 5 min for setting the temperature before data acquisition (the waiting time may be set via automation). Automatic tuning and matching of the probe was carried out for each sample. Samples were not spun. Standard shim settings from a saved file were loaded with each new sample. MeOD signal (from the extraction solution) was used for the field/frequency lock and shims were optimized for each sample using the TopShim program for gradient shimming (Z1 to Z6) followed by trimming of (Z1, Z2, X, Y) shims. The 90° pulse length was determined for each sample with pulsecal module. These three last steps can be

managed by au-program, au_metabo, downloadable on GitHUB (https://github.com/INRA/AU_metabo).

^{1}H -NMR spectra were obtained using a classic water suppression pulse sequence (90° proton pulse) with a scan duration of 20 s. Each spectrum consisted of 4 dummy scans and 32 scans of 128K data points. Instrument specific acquisition parameters are summarized in the Table S3.1.

Table S3.1. Instrument specific acquisition parameters

Instrument	Pulse sequence	Relaxation delay (s)	Acquisition time (s)	Scan duration (AQ + d1) (s)	Spectral window (ppm)	Pulse width	Receiver gain
JEOL 400 MHz	Proton with presaturation	10.64	9.36	20	14	90° calculated for each sample	Fixed (50)
Bruker 500 MHz	zgpr	10.60	9.40	20	14	90° calculated for each sample	Fixed (406)
Bruker 600 MHz	zgpr	12.20	7.80	20	14	90° calculated for each sample	Fixed (128)
