# Supplementary material for: Optimizing 1D 1H-NMR profiling of plant samples for high throughput analysis: extract preparation, standardization, automation and spectra processing
Source: Metabolomics. 2019 Feb 26;15(3):28. doi: 10.1007/s11306-019-1488-3 (PMC6394467; doi:10.1007/s11306-019-1488-3)
Supplement: Supplementary file 4 — Supplementary material 4 (PDF 329 KB) [file 11306_2019_1488_MOESM4_ESM.pdf]

Journal: Metabolomics
Title: Optimizing 1D ^{1}H-NMR profiling of plant samples for high throughput analysis: extract preparation, standardization, automation and spectra processing
Authors: Catherine Deborde, Jean-Xavier Fontaine, Daniel Jacob, Adolfo Botana, Valérie Nicaise, Florence Richard-Forget, Sylvain Lecomte, Cédric Decourtil, Kamar Hamade, François Mesnard, Annick Moing, Roland Molinié

Online resource 4. Examples of pH range and EDTA-*d12* needed for pH adjustment and paramagnetic cation chelation for preparation of plant tissue semi-polar methanolic extracts. Seven types of plant sample sets are used.

Tissue	Number of plant samples (biological replicates)	Plant powder weight (mg DW)	Volume of extract (µl)	Plant extract $pH_{apparent}$ before adjustment			Plant extract $pH_{apparent}$ after adjustment	Volume of NaOD 1M added (µl)	Volume of DCl 1 M added (µl)	NMR tube	
				Min	Max	Mean				Extract volume (µl)	EDTA-*d12* concentration (mM)
Eggplant leaf	23	40	750	5.54	6.35	5.96 ± 0.2	6.00 ± 0.01	1.38 ± 0.94	2.34 ± 0.49	700	9.3
White oak leaf	20	40	800	4.86	5.96	5.18 ± 0.24	6.00 ± 0.01	4.32 ± 2.69	4.03 ± 8.02	800	10.2
Wheat spikelet	60	30	900	6.39	6.63	6.50 ± 0.06	6.00 ± 0.01	0.09 ± 0.16	5.64 ± 0.72	700	9.4
Tomato pericarp (unripe or ripe)	36	40	900	5.08	6.16	5.72 ± 0.31	6.00 ± 0.01	2.55 ± 2.83	0.54 ± 0.50	800	11.8
Wild tomato ripe fruit pericarp	24	40	670	4.82	5.81	5.33 ± 0.26	6.00 ± 0.01	5.59 ± 3.60	0.22 ± 0.28	630	11.5
Sweet pepper ripe fruit	24	40	670	5.83	6.36	6.03 ± 0.13	6.00 ± 0.01	1.15 ± 2.82	2.52 ± 5.0	630	11.5
Flax root	80	30	800	5.70	6.79	6.07 ± 0.18	6.00 ± 0.01	0.13 ± 0.05	2.02 ± 1.9	800	16.0
