# Supplementary material for: Optimizing 1D 1H-NMR profiling of plant samples for high throughput analysis: extract preparation, standardization, automation and spectra processing
Source: Metabolomics. 2019 Feb 26;15(3):28. doi: 10.1007/s11306-019-1488-3 (PMC6394467; doi:10.1007/s11306-019-1488-3)
Supplement: Supplementary file 5 — Supplementary material 5 (PDF 701 KB) [file 11306_2019_1488_MOESM5_ESM.pdf]

Journal: Metabolomics
Title: Optimizing 1D ^{1}H-NMR profiling of plant samples for high throughput analysis: extract preparation, standardization, automation and spectra processing
Authors: Catherine Deborde, Jean-Xavier Fontaine, Daniel Jacob, Adolfo Botana, Valérie Nicaise, Florence Richard-Forget, Sylvain Lecomte, Cédric Decourtil, Kamar Hamade, François Mesnard, Annick Moing, Roland Molinié

Online resource 5. Spectra and data processing methods

5.1 AU program for full width at half maximum (FWHM)

After the time domain signals obtained were Fourier transformed with a 0.3 Hz exponential line broadening factor and zero filled (X2) to give spectra with 256K points with TopSpin and the resulting ^{1}H spectra were automatically phased and calibrated at 0 ppm to TMSP signal using TopSpin, spectra quality criteria were measured.
The determination of the two selected spectra quality criteria full width at half maximum (FWHM) for TMSP and CHD_2OD resonances were obtained with the au_TMSPMeOH program, downloadable on GitHUB (https://github.com/INRA/AU_metabo).

5.2 NMRProcFlow

For the examples presented in the present tutorial, all the spectra processing steps mentioned above were performed using the NMRProcFlow web application (Jacob et al. 2017) that provides a complete set of tools for processing and visualizing 1D NMR data recorded on Bruker, JEOL or Varian/Agilent spectrometers, within an interactive interface based on spectra visualization.

- Step 1: Pre-processing
  The time domain signals obtained were Fourier transformed with a 0.3 Hz exponential line broadening factor and zero filled (X2) to give spectra with 128K points and the resulting ^{1}H spectra were automatically phased and calibrated at 0 ppm to TMSP signal.

- Step 2: Baseline corrections
  Baseline corrections were all based on the airPLS (adaptive iteratively reweighted penalized least squares) algorithm (Zhang et al., 2010). The chosen criterion for the four spectra sets (JEOL400, Bruker500 & Bruker600) were to decrease the contribution of macromolecules (**Fig OR5.1**). Indeed, these latter could introduce a bias in variances (intra & inter groups) thus distorting any comparison between spectra sets.

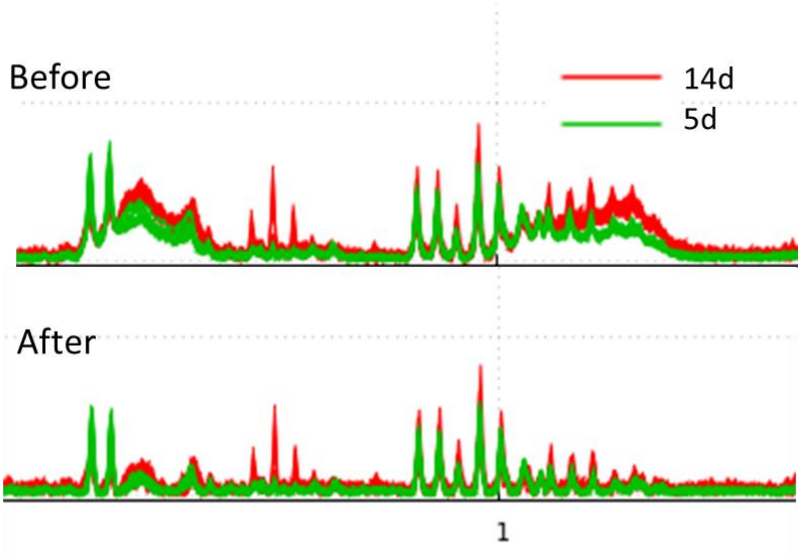

Fig OR5.1 - Example of baseline correction upon the JEOL400 spectra set within the ppm range [0.76 – 1.82]. *Top* : before and *Bottom* : after the baseline correction.

- Step 3: Spectra alignment

  Realignment was performed with a novel peak alignment algorithm, called hierarchical Cluster-based Peak Alignment (CluPA), proposed by Vu et al (2011) implemented in NMRProcFlow. Given that the CluPA method allows to align ppm areas with many peaks, we have applied a realignment for 3 different spectral zones according to the type of compound resonances within these zones (0 up 1 ppm for Aliphatics, 1 up to 3 for Organic acids, 3 up to 5.5 for Sugars). No realignment has been performed in the Aromatic zone (5.5 up to 10). See **Fig OR5.2** (*top & middle*) for a realignment within the organic acid resonance spectral zone.

- Step 4: Bucketing

  We performed the bucketing based on the Adaptive, Intelligent Binning method (De Meyer et al. 2008). This method attempts to split the spectra so that each area common to all spectra contains the same single resonance, i.e. belonging to the same metabolite (Intelligent bucketing) thus producing more relevant variables (**Fig OR5.2** *bottom*)

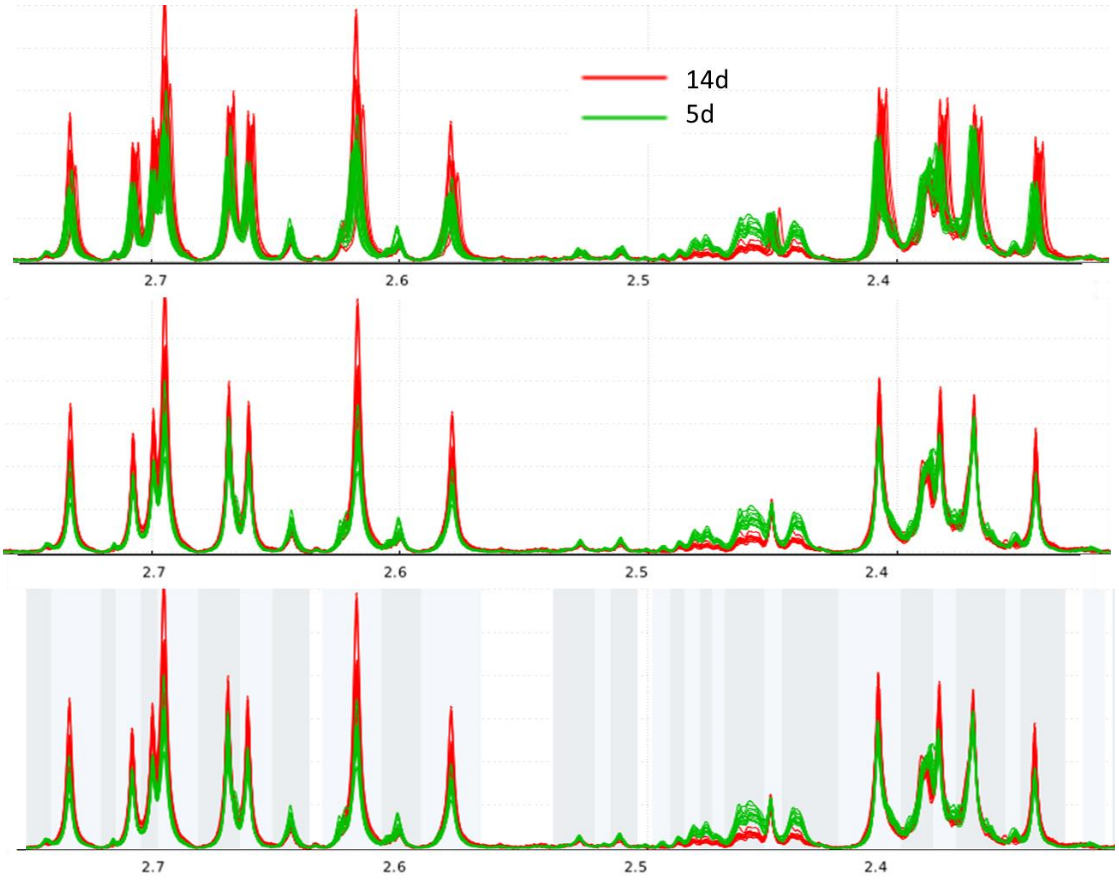

Fig OR5.2 Example of alignment then bucketing upon the JEOL400 spectra within the ppm range [2.30 - 2.75]. *Top* : Before and *Middle* : After spectra alignment. *Bottom* : Intelligent Bucketing was applied.

References

De Meyer, T., Sinnaeve, D., Van Gasse, B., Tsiporkova, E., Rietzschel, E. R., De Buyzere, M. L., et al. (2008). NMR-based characterization of metabolic alterations in hypertension using an adaptive, intelligent binning algorithm. *Analytical Chemistry*, 80(10), 3783–3790, doi: 10.1021/ac7025964

Jacob, D., Deborde, C., Lefebvre, M., Maucourt, M. and Moing, A. (2017) NMRProcFlow: A graphical and interactive tool dedicated to 1D spectra processing for NMR-based metabolomics. *Metabolomics*, 13, 36, doi:10.1007/s11306-017-1178-y

Vu T.N., Valkenborg D., Smets K., Verwaest K.A., Dommisse R., Lemière F., Verschoren A., Goethals B., Laukens K. (2011). An integrated workflow for robust alignment and simplified quantitative analysis of NMR spectrometry data. *BMC Bioinformatics,* 12, 405, doi: 10.1186/1471-2105-12-405

Zhang Z, Chen S, and Liang Y-Z (2010) Baseline correction using adaptive iteratively reweighted penalized least squares. *Analyst*, 2010, 135, 1138-1146, doi:10.1039/B922045C
