# Supplementary material for: Optimizing 1D 1H-NMR profiling of plant samples for high throughput analysis: extract preparation, standardization, automation and spectra processing
Source: Metabolomics. 2019 Feb 26;15(3):28. doi: 10.1007/s11306-019-1488-3 (PMC6394467; doi:10.1007/s11306-019-1488-3)
Supplement: Supplementary file 6 — Supplementary material 6 (PDF 435 KB) [file 11306_2019_1488_MOESM6_ESM.pdf]

Journal: Metabolomics
Title: Optimizing 1D ^{1}H-NMR profiling of plant samples for high throughput analysis: extract preparation, standardization, automation and spectra processing
Authors: Catherine Deborde, Jean-Xavier Fontaine, Daniel Jacob, Adolfo Botana, Valérie Nicaise, Florence Richard-Forget, Sylvain Lecomte, Cédric Decourtil, Kamar Hamade, François Mesnard, Annick Moing, Roland Molinié

Online resource 6. Comparison of spectra of Bruker (600 and 500 MHz) and JEOL (500 and 400 MHz) instruments.

Representative spectra portions of a given semi-polar methanolic extract of wheat spikelet at 5 DAF acquired on each instrument. Blue, Bruker 600 MHz; Purple, Bruker 500 MHz; Red, JEOL 500 MHz; Green, JEOL 600 MHz. The 500 MHz JEOL spectrometer was not equipped with an autosampler, therefore only a few wheat spikelet extracts were measured with it.

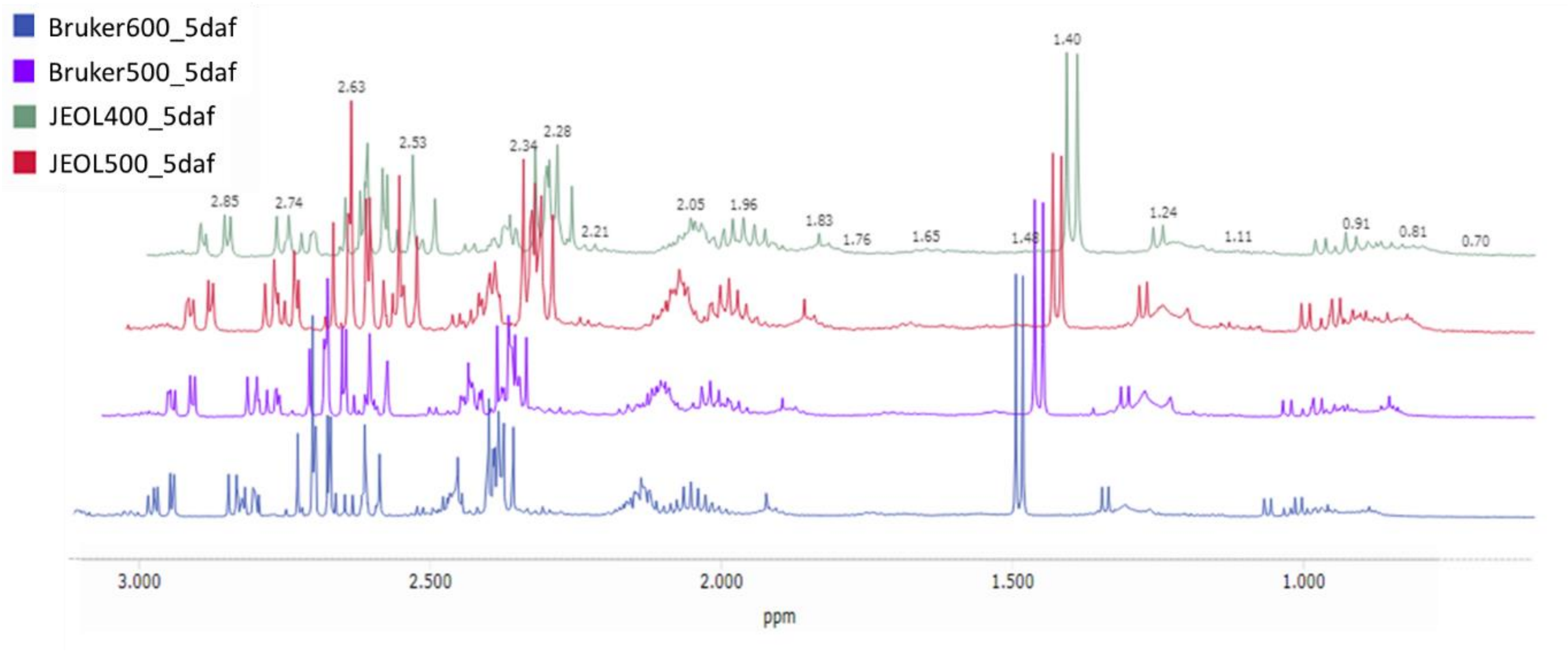

Fig OR6.1 Comparison of spectra of Bruker (600 and 500 MHz) and JEOL (500 and 400 MHz) instruments
