# Supplementary material for: Optimizing 1D 1H-NMR profiling of plant samples for high throughput analysis: extract preparation, standardization, automation and spectra processing
Source: Metabolomics. 2019 Feb 26;15(3):28. doi: 10.1007/s11306-019-1488-3 (PMC6394467; doi:10.1007/s11306-019-1488-3)
Supplement: Supplementary file 7 — Supplementary material 7 (PDF 956 KB) [file 11306_2019_1488_MOESM7_ESM.pdf]

Journal: Metabolomics
Title: Optimizing 1D ^{1}H-NMR profiling of plant samples for high throughput analysis: extract preparation, standardization, automation and spectra processing
Authors: Catherine Deborde, Jean-Xavier Fontaine, Daniel Jacob, Adolfo Botana, Valérie Nicaise, Florence Richard-Forget, Sylvain Lecomte, Cédric Decourtil, Kamar Hamade, François Mesnard, Annick Moing, Roland Molinié

Online resource 7. PCA details of the wheat sample set and clustering comparison of 600 and 500 MHz Bruker and 400 MHz JEOL instruments.

Each of the three data matrices comprises the buckets issued from 22 ^{1}H NMR spectra of spikelet extracts (5 or 14 DAF) as described in Online Resource 5.

To pave the way for detailed biological interpretation of PCA loadings, which is not the purpose of this tutorial, we performed a clustering analysis based on a threshold applied on the bucket correlation matrix (Jacob et al. 2013). Then we performed an automatic cluster matching (equivalent to compound signatures) from a bank of 86 ^{1}H NMR spectra of reference compounds (Solvent D_2O, $pH_{apparent}$ 6, 500 MHz) (Jacob et al. 2013 & Jacob et al. 2018).

On the basis of the lowest resolution (JEOL400), we were able to automatically identify 9 compounds (Alanine, Asparagine, Aspartate, Citric acid, Glucose, Glutamine, Malic acid, Phenylalanine and Sucrose). (**Fig OR7.1**).

**Compound**	**KEGG ID**	**MSI level**
Alanine	C01401	1
Asparagine	C16438	1
Aspartate	C16433	1
Citric acid	C00158	1
Glucose	C00031	1
Glutamine	C00303	1
Malic acid	C00711	1
Phenylalanine	C02057	1
Sucrose	C00089	1

We processed in the same way for Bruker500 (**Fig OR7.2**) and Bruker600 (**Fig OR7.3**), but we only kept the annotations for the 9 compounds previously identified for the JEOL400 spectra.

	# Buckets	# Clusters	# Clusterized Buckets	Correlation threshold
JEOL 400	245	20	84	0.945 +/- 0.01
Bruker 500	397	34	131	0.9575 +/- 0.01
Bruker 600	465	40	137	0.974 +/- 0.01

Table OR7.1: Number of buckets obtained after intelligent bucketing by means of NMRProcFlow and distribution of bucket clusters for the wheat spikelet dataset for the three NMR instruments.

JEOL 400 MHz

```
#-- Clustering --
#   Correlation Method: pearson
#   Correlation Threshold : 0.945
#   Correlation Tolerance: 0.01
#   Nb Clusters: 20
#   Clustered variables: 84 / 245
```

Cluster	Compound	PC1	PC2
C8	Alanine	-0.0660	-0.0816
C3	Asparagine	-0.0829	0.0435
C7	Aspartate	-0.0654	0.1128
C5	Citric acid	0.0693	0.0179
C4	Glucose	-0.0836	-0.0006
C2	Glutamine	-0.0849	0.0405
C1	Malic acid	0.0864	0.0378
C20	Phenylalanine	0.0700	0.0421
C13	Sucrose	0.0655	-0.1198

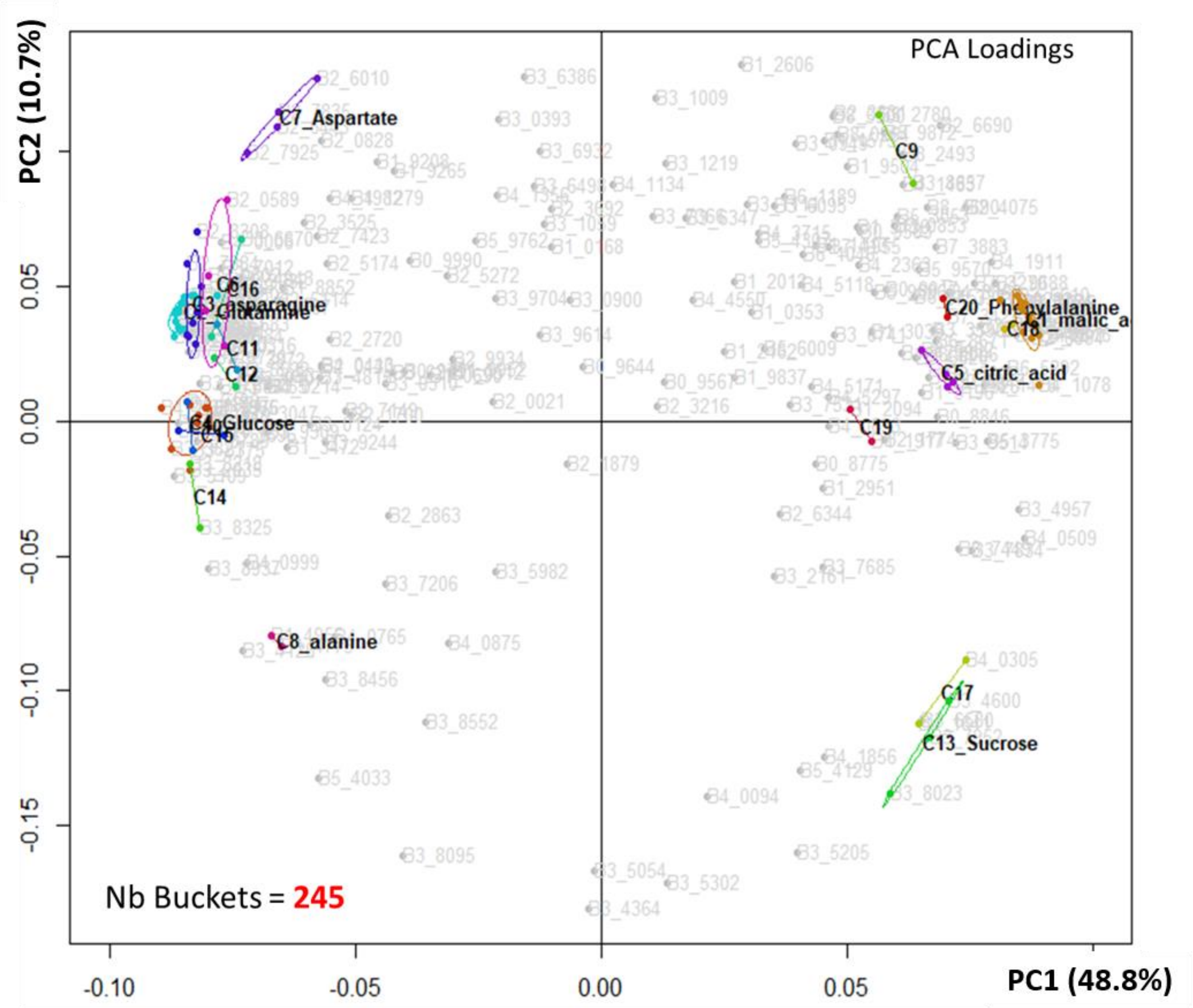

Fig OR7.1 Loadings plot of the PCA applied on the JEOL400 dataset (*Bottom*)- The clusters generated by applying a threshold to the correlation matrix have been added. (*Top left*: clustering parameter values). The annotated clusters correspond to the selected compounds (*Top right*: the list of annotated clusters with their coordinates on the loading plot).

Bruker 500 MHz

```
#-- Clustering --
#   Correlation Method: pearson
#   Correlation Threshold : 0.9575
#   Correlation Tolerance: 0.01
#   Nb Clusters: 34
#   Clustered variables: 131 / 397
```

Cluster	Compound	PC1	PC2
C13	Alanine	-0.0527	-0.0729
C3	Asparagine	-0.0660	0.0114
C17	Aspartate	-0.0542	0.0844
C16	Citric acid	0.0489	0.0034
C1	Glucose	-0.0656	0.0192
C2	Glutamine	-0.0652	0.0395
C7	Malic acid	0.0603	0.0483
C29	Phenylalanine	0.0501	0.0569
C23	Sucrose	0.0427	-0.0998

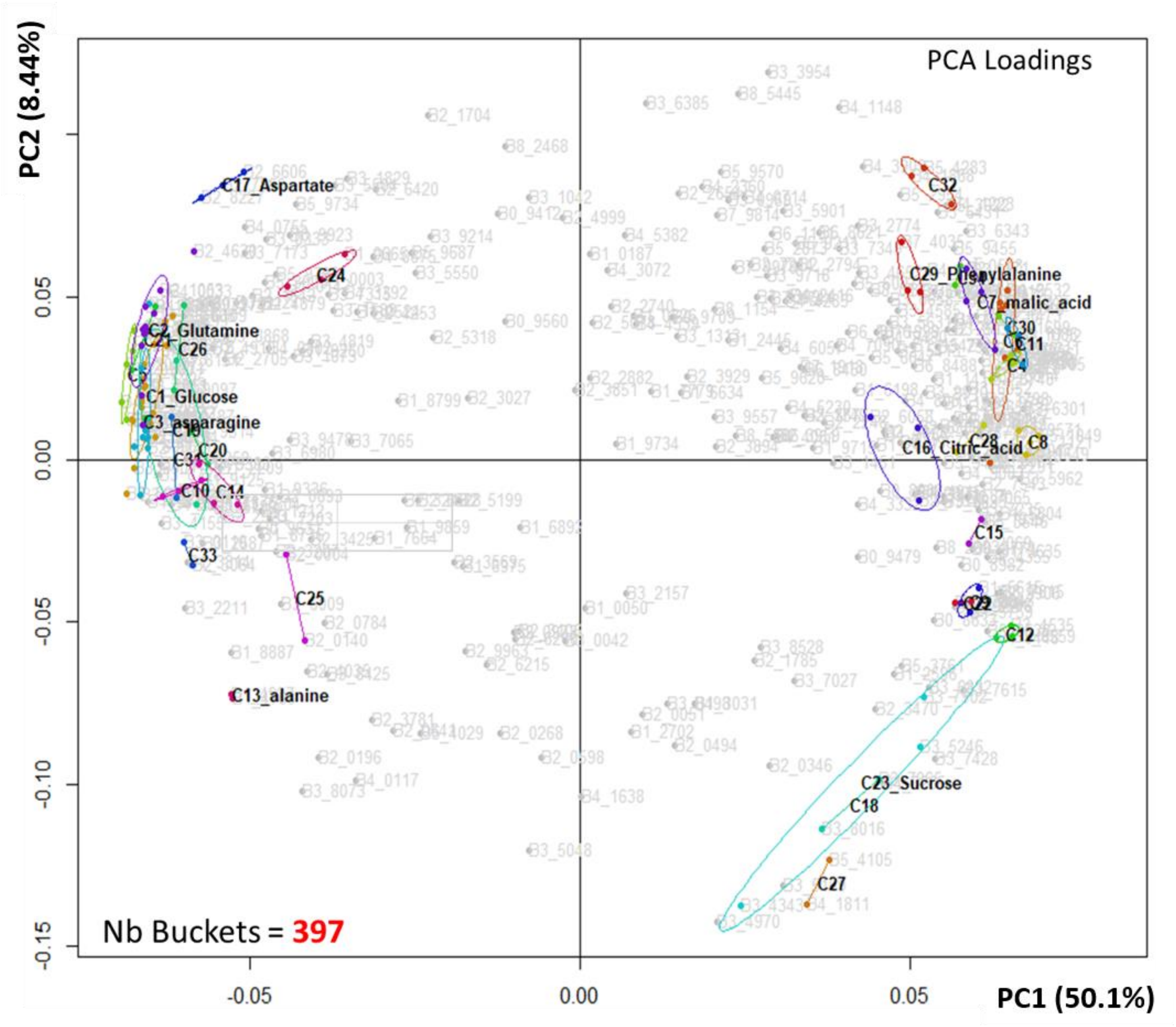

Fig OR7.2 Loadings plot of the PCA applied on the Bruker500 dataset (*Bottom*)- The clusters generated by applying a threshold to the correlation matrix have been added. (*Top left*: clustering parameter values). The annotated clusters correspond to the selected compounds (*Top right*: the list of annotated clusters with their coordinates on the loading plot).

Bruker 600 MHz

```
#-- Clustering --
#   Correlation Method: pearson
#   Correlation Threshold : 0.974
#   Correlation Tolerance: 0.01
#   Nb Clusters: 40
#   Clustered variables: 137 / 465
```

Cluster	Compound	PC1	PC2
C12	Alanine	-0.0496	-0.0585
C3	Asparagine	-0.0600	0.0269
C5	Aspartate	-0.0474	0.0835
C8	Citric acid	0.0518	0.0084
C2	Glucose	-0.0594	0.0096
C1	Glutamine	-0.0607	0.0266
C4	Malic acid	0.0612	0.0242
C29	Phenylalanine	0.0587	0.0361
C28	Sucrose	0.0549	-0.0728
C36	Sucrose	0.0423	-0.1103

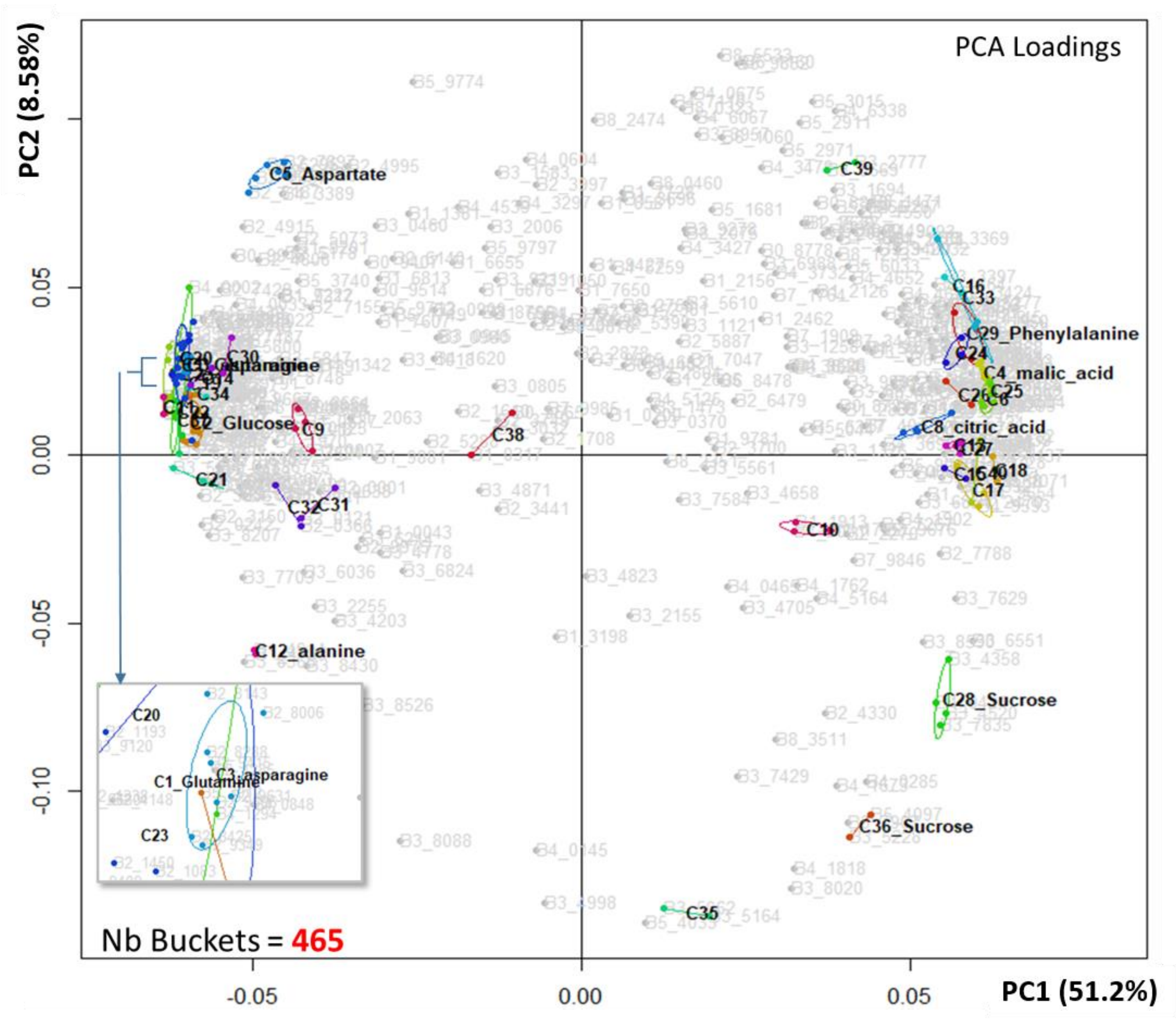

Fig OR7.3 Loadings plot of the PCA applied on the Bruker600 dataset (*Bottom*)- The clusters generated by applying a threshold to the correlation matrix have been added. (*Top left*: clustering parameter values). The annotated clusters correspond to the selected compounds (*Top right*: the list of annotated clusters with their coordinates on the loading plot).

References

Jacob, D., Deborde, C., & Moing A. (2013). An efficient spectra processing method for metabolite identification from 1H-NMR metabolomics data. *Analytical and Bioanalytical Chemistry,* 405(15), 5049–5061, doi: 10.1007/s00216-013-6852-y

Jacob, D., Deborde, C., & Lefebvre, M. (2018). Rnmr1D: Perform the Complete Processing of a Set of Proton Nuclear Magnetic Resonance Spectra. R package version 1.2.1. https://cran.r-project.org/package=Rnmr1D
